# How Human Activities Affect Groundwater Storage

**DOI:** 10.34133/research.0369

**Published:** 2024-05-29

**Authors:** Ying Zhao, Meiling Zhang, Zhuqing Liu, Jiabin Ma, Fan Yang, Huaming Guo, Qiang Fu

**Affiliations:** ^1^ School of Water Conservancy & Civil Engineering, Northeast Agricultural University, Harbin 150030, China.; ^2^ International Cooperation Joint Laboratory of Health in Cold Region Black Soil Habitat of the Ministry of Education, Harbin 150030, China.; ^3^ Ministry of Education Key Laboratory of Groundwater Circulation and Environmental Evolution & School of Water Resources and Environment, China University of Geosciences (Beijing), Beijing 100083, China.

## Abstract

Despite the recognized influence of natural factors on groundwater, the impact of human activities remains less explored because of the challenges in measuring such effects. To address this gap, our study proposes an approach that considers carbon emissions as an indicator of human activity intensity and quantifies their impact on groundwater storage. The combination of carbon emission data and groundwater storage data for 17,152 grid cells over 16 years in 4 typical basins shows that they were generally negatively correlated, whereas both agriculture and aviation had positive impacts on groundwater storage. The longest impact from aviation and agriculture can even persist for 7 years. Furthermore, an increase of 1 Yg CO_2_/km^2^ per second in emissions from petroleum processing demonstrates the most pronounced loss of groundwater storage in the Yangtze River Basin (approximately 4.1 mm). Moreover, regions characterized by high-quality economic development tend to have favorable conditions for groundwater storage. Overall, our findings revealed the substantial role of human activities in influencing groundwater dynamics from both temporal and spatial aspects. This study fills a crucial gap by exploring the relationship between human activities and groundwater storage through the introduction of a quantitative modeling framework based on carbon emissions. It also provides insights for facilitating empirical groundwater management planning and achieving optimal emission reduction levels.

## Introduction

Groundwater is a crucial resource that serves as the primary water source for more than 2 billion people [1]. However, groundwater is often overlooked by humans due to its invisibility. Most urgently, groundwater storage (GWS) is steadily decreasing in many regions owing to climate change, inappropriate human use, and insufficient long-term management [2]. The decline of the groundwater table constrains economic development [3] and increases the risk of saltwater intrusion and contamination [4]. More than half of the world’s groundwater-extracting basins are expected to face depletion owing to uncontrolled human extraction by 2050 [5], with natural replenishment not able to keep pace with extraction rates, necessitating immediate action [5,6]. However, determining the extent to which society should limit the impact of human activities on groundwater is a great challenge for policymakers. Therefore, balancing human activities and groundwater sustainability based on quantifying the impact of human activities on GWS is the primary issue to be addressed in hydrology today.

Currently, numerous studies have significantly advanced our understanding of how human activities and climate change affect GWS [7]. Notably, anthropogenic groundwater extraction, such as agricultural irrigation, industrial, and other sectors, plays a major role in depleting GWS than climate impact [6,8,9]. Despite the increasing number of studies on the societal impacts of groundwater depletion, they predominantly focused on agriculture or industry [10], while studies on examining the quantitative effects and comparative analyses of multiple human activities that utilize groundwater are lacking. Since the Industrial Revolution, fossil fuels have been widely used in all aspects of human society [11,12]. This transition has led to an increase in carbon emissions (CEs), with human activities identified as a major contributor [13–15], contributing to the greenhouse effect [16,17] and increasing the risk of extreme weather events [18,19] and natural disasters [20–22]. Thus, to mitigate these impacts and strive to achieve the Paris Agreement ambitious goal—which aims to limit global temperature rise to 1.5 °C above preindustrial levels—the cooperation and commitment of global food systems, forest systems, energy, and other sectors are imperative [23]. However, the specific effects of these widespread changes in human activity on groundwater resources remain largely unexplored. In addition, current groundwater management, characterized by sector-specific strategies, fosters competition rather than collaboration, endangering the sustainability of groundwater resources [24,25]. Therefore, this study proposes CE as an important indicator of human activity intensity. This proposal will contribute to facilitating a deeper understanding of the interplay between human activities and groundwater sustainability and provide support for the development of integrated strategies to harmonize groundwater management with human progress.

This study proposes that there are certain nonnegligible connections between CE, groundwater, and human activities, related to regional and specific emission sectors. This connection will be a key factor in reducing the burden on Earth in response to achieving net zero. Thus, to fully understand the impact of human activities on groundwater, this study collects historical CE and groundwater data in 4 representative basins with a small range of latitudinal variability, aiming to minimize climate impacts. The Yangtze River Basin (YRB), the Pearl River Basin (PRB), the Great Lakes Basin (GLB), and the Rhine Basin (RB) were selected, with 17,152 grid cells from 2003 to 2018. This study firstly explores the connection and related relationship between CE per capita (CEP) and GWS per capita (GWSP) using grey relational analysis and correlation analysis. Then, 2-factor fixed-effects panel regression models are applied to demonstrate the relationship and choose the best model in each basin for further analysis. Moreover, this study explores and considers spatial heterogeneity and long-term and short-term effects.

## Results and Discussion

**Fig. 1.**
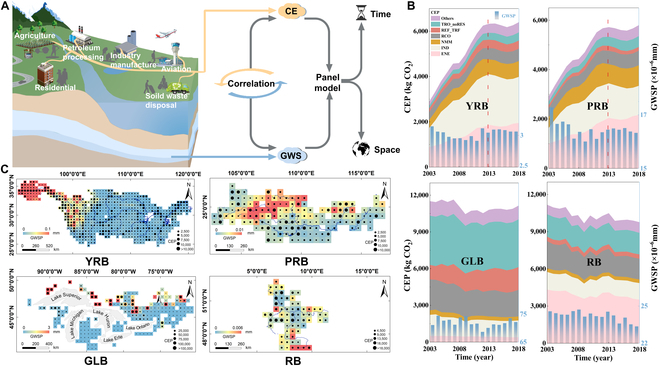
(A) Processes involved in human activities through CE impact GWS. (B) Time trends of CEP (in kilograms of CO_2_) from energy industry (ENE), combustion for manufacturing (IND), production of nonmetallic minerals (NMM), residential (RCO), oil refineries and transformation industry (REF_TRF), road transport (TRO_noRES), and GWSP (in millimeters) from 2003 to 2018. (C) Distributions of total CEP and GWSP in 2018 of 4 basins.

The influence of human activities on groundwater has long posed a substantial challenge within hydrological studies. It is an undeniable fact that human activities produce CE [26,27]. Therefore, Fig. 1A illustrates the approach used in this study. The temporal variations of CEP and GWSP from 2003 to 2018 in the 4 basins are shown in Fig. 1B. Specifically, CEP showed a slow upward trend in the YRB and PRB before 2013 and remained stable thereafter, whereas it was more volatile in the GLB and RB. GWSP remained relatively stable in recent years but differed significantly among the 4 basins. The spatial distributions of CEP and GWSP, as illustrated in Fig. 1C, show that in the 4 basins, GWSP was more abundant upstream and in areas near lakes, whereas CEP was higher in areas through which rivers flowed and near lakes. The specific correlation between the two and the spatial and temporal connection are introduced in the next section. The spatial distributions of CEP and GWSP over time are shown in Fig. S3.

### Correlation between CEP and GWSP

**Fig. 2.**
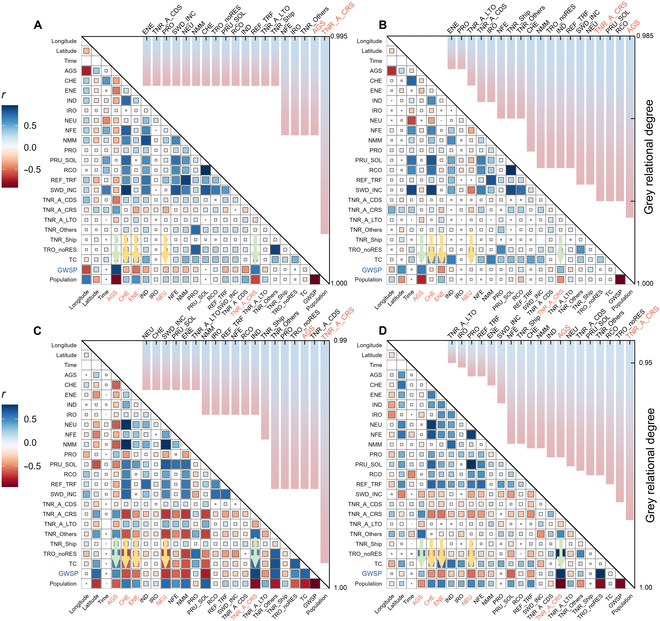
Grey relational response figure (top-right corner) and heatmap of correlation analysis (bottom-left corner) for various sectors of CEP and GWSP in 4 basins. (A) YRB, (B) PRB, (C) GLB, and (D) RB. The abbreviations are shown in Table S1.

Figure 2 shows the connections and importance rankings of various sectors of CEP and GWSP. The results showed that CEP for all sectors has a marked connection with GWSP, with all grey relational degrees exceeding 0.95. Aviation (TNR_A_CRS) and agricultural emissions (AGS) showed the strongest connection with GWSP, whereas energy emissions exhibited a relatively weaker connection. The results of the grey relational analysis suggest that various human activities are indeed closely related to GWSP, especially aviation and agriculture, which play an important role in the dynamic changes in GWSP. This pattern was evident in all the 4 typical basins.

Furthermore, this study explored the correlation between various sectors of CE and GWS and those of CEP and GWSP. The impact of CE on GWS was predominantly negative in the PRB, GLB, and RB but exhibited significant positive effects in the YRB (Fig. S2). This reveals that groundwater is used in almost all sectors in PRB, GLB, and RB, whereas this is not reflected in the YRB due to the greater complexity. Compared to the other 3 basins, YRB spans a wider range of latitudes (approximately 10°), featuring more complex climates and geographical environments. The secondary reason is the more concentrated population distribution and the substantial disparity in population size compared to the other 3 basins (Fig. 5 and Fig. S3A and B background). Figure 2 also demonstrates that GWSP was significantly correlated with CEP. Specifically, energy industry (ENE), chemical production (CHE), and petroleum processing (NEU) emissions were largely negatively correlated with GWSP (Fig. 2 and Table S1). Negative correlations indicated that the energy industry, chemical production, and petroleum processing sectors consumed large amounts of groundwater. These sectors should be key focus for protecting groundwater resources in the future. However, agriculture and aviation emissions exhibited a strong positive correlation with GWSP (Fig. 2 and Table S1). The positive correlation of agriculture demonstrates that surface water may be involved in irrigation in the 4 basins, not just groundwater pumping. Agriculture is routinely thought to reduce GWS [28,29]; however, no significant negative effects were observed in these 4 basins. Taking the YRB as an example, in 2021, the surface water supply was 200.362 billion m^3^, far exceeding the total groundwater supply of 3.988 billion m^3^, while agricultural water usage reaching 103.090 billion m^3^ [30]. Therefore, in areas with abundant surface water, agricultural irrigation may contribute to the increase in groundwater through infiltration [31], differing from the common situation of excessive groundwater consumption in agriculture. The positive correlation in the aviation sector suggests that areas with high economic levels (high aviation CEP) contribute to the sustainability of GWSP [32–34], which will be discussed in detail in the “Effects of spatial CEP heterogeneity on GWSP” section.

For each specific basin, GWSP responded differently to CEP. There was a stronger correlation in the GLB and a weaker correlation in the PRB, demonstrating that various sectors in the GLB rely heavily on groundwater, but those in the PRB are opposite. To explore the temporal and spatial associations of specific emission sectors in specific basins, we use 2-factor fixed-effects panel regression models for further analysis using the CEP and GWSP data.

### Impact of CEP on GWSP by 2-factor fixed-effects panel regression models

The influences of various sectors of CEP on GWSP, in terms of both temporal sequences and more detailed spatial scales, are currently unknown. However, these impacts are of significant importance for targeted CE reduction and groundwater utilization balance. This research used panel regression models to investigate the spatiotemporal effects of CEP on GWSP and chose the best accuracy model as the foundational model for subsequent analysis in each basin by comparing the adjusted coefficient of determination (adjusted *R*^2^) and Akaike information criterion (AIC) [Table and Tables S2, model (4)].

**Table.** Regression results from different panel model specifications for CEP on GWSP in 4 basins

Basin	YRB	PRB	GLB	RB
CEP sector				
AGS	0.084 (0.002)***	0.003 (2 × 10^−4^)***	0.038 (0.01)***	−0.001 (3 × 10^−4^)***
CHE		0.000 (2 × 10^−5^)**		0.000 (7 × 10^−5^)***
IRO		−0.001 (9 × 10^−5^)***		
PRU_SOL^a^	7.037 (0.567)***	0.006 (0.002)***	−3.111 (0.56)***	
ENE		0.000 (6 × 10^−7^)**		
IND		0.000 (4 × 10^−6^)***	0.011 (0)***	
NEU^a^	−4.101 (0.558)***	−0.009 (0.002)***		−0.007 (0.001)***
NMM		0.000 (3 × 10^−6^)***		0.000 (5 × 10^−5^)***
PRO		0.000 (1 × 10^−4^)***		
RCO	−0.241 (0.017)***	0.000 (5 × 10^−5^)***	0.079 (0.01)***	0.000 (1 × 10^−5^)***
REF_TRF		0.000 (6 × 10^−6^)***	0.041 (0.01)**	
TNR_A_CRS	0.070 (3 × 10^−4^)***	0.000 (5 × 10^−5^)***	0.011 (0)***	0.001 (7 × 10^−5^)***
TNR_A_LTO		0.002 (3 × 10^−4^)***		
TNR_Others	−0.016 (0.003)***	0.000 (7 × 10^−5^)*	−0.004 (0)***	0.001 (2 × 10^−4^)***
TNR_Ship	0.000 (5 × 10^−5^)***	0.000 (1 × 10^−5^)***		
TRO_noRES			0.000 (0)*	0.000 (6 × 10^−6^)***
SWD_INC		−0.085 (0.006)***		
Adjusted *R*^2^	0.935	0.541	0.964	0.603
*N*	10,400	2,448	3,232	1,072
AIC	19,853.62	−33,170.80	20,846.80	−12,867.38

The coefficients from the regression models for the 4 basins are shown. The coefficients estimated the effect of a one-unit change in CEP on GWSP change. Standard errors are shown in parentheses. *P < 0.05, **P < 0.01, and ***P < 0.001.

^a^ The 2 largest marginal impacts in the 4 basins.

Specifically, an increase in one unit (in yottagrams of CO_2_ per square kilometer per second) of industrial manufacturing (PRU_SOL) CEP was accompanied by an even 7-mm increase in GWSP in the YRB, hereafter referred to as the marginal benefits [35]. However, in GLB, each additional unit of industrial manufacturing CEP resulted in a loss of 3 mm in GWSP, hereafter referred to as the marginal losses. In terms of petroleum processing, one unit increase was accompanied by a maximum decrease of 4.1 mm in GWSP in the YRB. The increase in agricultural emissions was typically accompanied by an increase in GWSP in majority basins (except the RB). This suggests that not only the energy sector is the one that needs attention but also industrial manufacturing may be important for maintaining GWSP.

Industrial manufacturing emissions were found to contribute significantly to the marginal benefit of GWSP in the YRB and PRB, implying that surface water in product manufacturing and industrial processes plays an important role in groundwater recharge. However, the impact of industrial manufacturing CEP on GWSP was diametrically opposed in the GLB, showing that industrial activities have a greater negative impact on GWS than other CE. This is supported by the fact that industrial manufacturing processes pump large quantities of groundwater for application in the GLB, whereas surface water is dominant in the YRB and PRB [36–38]. Petroleum processing is an important industry for reducing GWSP, particularly in the YRB, PRB, and RB. Oil extraction and processing (e.g., cooling, washing, and other processes) typically require the use of large amounts of surface water and groundwater [39]. It is estimated that the water consumption of China’s oil sector in 2019 was approximately 390 million tons [40]. In the United States, approximately 1.50 trillion gallons of water have been used in oil and gas production since 2011, much of which is from groundwater [41,42]. In fact, a previous study has shown that the carbon footprint in petroleum processing driven by exports is concentrated in Shanghai, Guangzhou, and Ningbo, which are located in the YRB and PRB [43,44]. Petroleum processing and mining have resulted in considerable groundwater extraction in the RB, to the extent that several groundwater bodies in the industrial zone have been classified as near-unsustainable extraction areas [45]. This means that some measures may need to be implemented to maintain a balance in groundwater use, such as the adoption of clean energy sources [46].

### Spatiotemporal impacts of CEP on GWSP

#### Long-term impact of CEP on GWSP

There is an ongoing debate as to whether the spatiotemporal impacts are persistent or instantaneous [47–50]. The persistent effects of CEP on GWSP have substantial implications for devising effective policies for rational groundwater extraction and mitigation of depletion in the long term. Following recent literature [51–53], we used distributed lag models to address the persistent effect of CEP on GWSP.

**Fig. 3.**
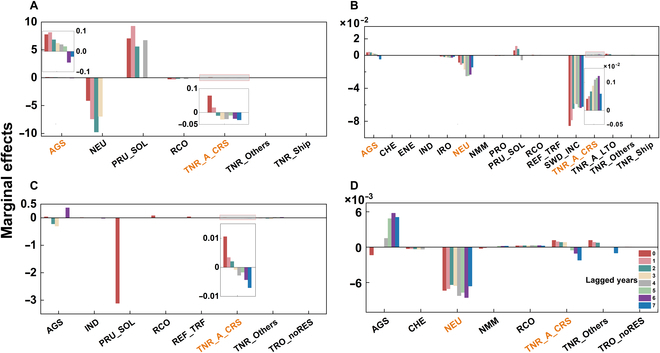
The delayed effect of CEP on GWSP from distributed lag models up to 7 lagged years in 4 basins. (A) YRB, (B) PRB, (C) GLB, and (D) RB. The color band indicates the lagged years.

Aviation, agricultural, and petroleum processing CEP had persistent impacts on GWSP that can last for even 7 years (Fig. 3). In contrast, the impact of industrial manufacturing emissions on the GWSP lasted only 2 years in the YRB and PRB.

Interestingly, both the agriculture and aviation CEP sectors exhibited a positive correlation, showing similar long-term impacts in both YRB and PRB. This suggests that prolonged and sustained water use in agriculture and aviation is beneficial for long-term groundwater recovery in these 2 basins. This could be related to the time required for the recharge of surface water through soil media into groundwater in aquifers [54]. The impacts were the greatest in the first 2 years.

Moreover, the impact can change from positive to negative in different years (Fig. 3). These results suggest that the effects of different emission sectors on GWSP vary in terms of duration and stability. Regardless of the persistence and stability issues, the impact of CEP on GWSP would have broader and more far-reaching unforeseen consequences, which is a very important guide for human decision-making.

#### Short-term impact of CEP on GWSP

The observed impact of the annual average CEP on GWSP mainly reflects the linear relationship between CEP and GWSP across the basins. However, the effect of different emissions on groundwater was reflected in the annual trend and in the interannual variations. To explore whether there are anomalies or trend changes in the growth of different emission sectors, this study considered the effect of variations in CEP on variations in GWSP. We established a measure and included it in the regression models: the variations in CEP and GWSP in the study areas relative to the previous year, named minus.

**Fig. 4.**
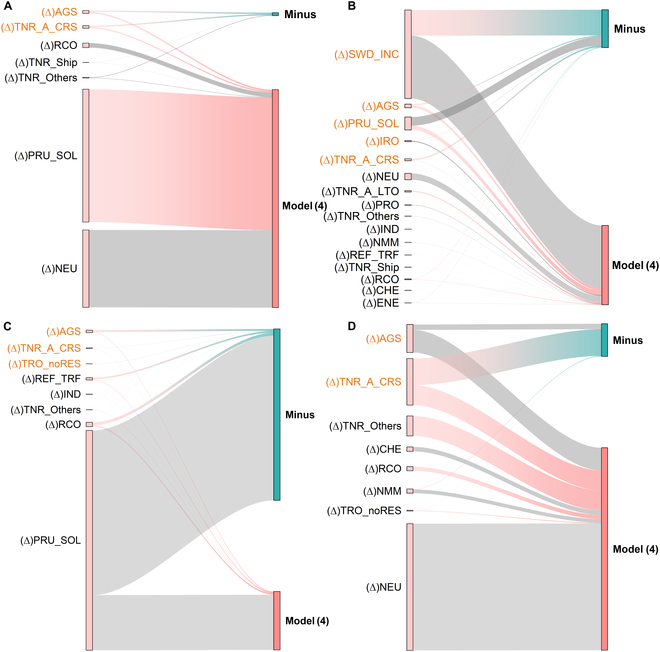
Marginal effects of each sector of CEP on GWSP for each basin. (A) YRB, (B) PRB, (C) GLB, and (D) RB. The left side of the subplot shows the level value (AGS) or variation [(∆)AGS] of each sector of CEP under the growth scenarios (minus); the thickness of the line indicates the size of the marginal effect; gray indicates the marginal losses; color indicates the marginal benefits. Sectors labeled in orange have short-term impacts.

Figure 4 shows that agricultural and aviation emissions had a short-term influence on GWSP in all 4 basins. Agricultural and aviation experienced more significant growth than other CEP sectors because of higher demand and faster rates. Agricultural and aviation significantly influenced GWSP at both long-term and short-term scales, which provide some insights into maintaining GWS from a sectoral perspective.

#### Effects of spatial CEP heterogeneity on GWSP

The impact of CEP on GWSP is influenced by its geographical distribution (Fig. S4). In the YRB, industrial manufacturing emissions had a negative effect on GWSP in the upper reaches and tributaries of the Yangtze River (that is, Qinghai Province, western Sichuan, Jiangxi Province, and southeastern Hunan; Fig. S4A, PRU_SOL). In contrast, areas with high populations in the middle and lower reaches of the YRB (i.e., eastern Sichuan, Chongqing, Hubei, Anhui, and Shanghai) had positive impacts on GWSP. This illustrates the unfavorable GWSP in the less industrialized upper reaches of the Yangtze, which are predominantly engaged in forestry and animal husbandry [55]. In the PRB, the impact of solid waste disposal (SWD_INC) emissions on GWSP was mainly negative upstream (Guizhou and Yunnan) and positive in the middle and downstream areas (Fujian), with greater marginal losses in areas along the river (Fig. S4B). In the GLB, industrial manufacturing emissions contribute to marginal losses to GWSP in the United States and marginal benefits in Canada (Fig. S4C, PRU_SOL). According to the World Resources Institute’s Water Stress Index, the water stress in Canada is low to medium, whereas that in the United States is medium to high. The United States is more likely to pump groundwater in these sectors, meaning that groundwater may decline with these CEP [56]. In the RB, the effects of petroleum processing emissions on GWSP were significantly negative in the northern part of the basin, specifically in the middle and lower reaches of the river (Germany and France; Fig. S4B). Conversely, the effect was positive in the upper reaches (Switzerland). These effects highlight the necessity for the development of region-specific groundwater management plans that integrate sectoral emissions control, land use planning, and cross-border cooperation. By focusing on the unique sectoral and contextual characteristics of each basin, policymakers and environmental managers can implement more effective and sustainable strategies for groundwater conservation.

**Fig. 5.**
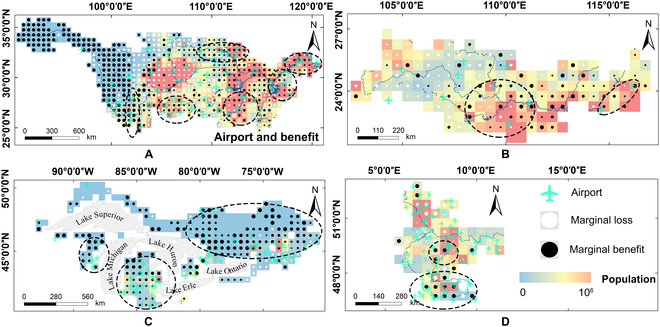
Distributions of the marginal effects of aviation CEP on GWSP in 4 basins. (A) YRB, (B) PRB, (C) GLB, and (D) RB. The blue and red color represents the population of that grid in 2018. The size of the dots indicates the strength of the marginal effects.

There was an interesting finding that aviation emissions were positively correlated with GWSP in all basins. Moreover, it has both short-term and long-term impacts. Thus, we further compared the spatial distribution of airports with marginal effects in the 4 basins. The results showed that areas with airports had a marginal benefit in most cases (Fig. 5). In the contemporary world, aviation is one of the most critical global economic activities, and places with high population densities are accompanied by more airports. The relationship between economic growth and aviation emissions is an important area of interest. Many studies have shown that economic growth is a major factor in increasing aviation emissions [57–59]. The more economically developed a region, the more airports it has. In turn, it contributes to the growth of the economy [60,61]. Economically developed regions tend to be dominated by high-value-added (e.g., high-tech) industries, tourism, and services and less by other CEP sectors, such as manufacturing [62,63], resulting in lower groundwater consumption. On the other hand, high-quality economic development has contributed to the emergence of social awareness regarding the critical importance of protecting groundwater resources. Related research demonstrates that airports are increasingly prioritizing sustainable water management practices globally [64,65]. Despite the increase in passenger traffic, water consumption for airport infrastructure and operations has decreased because of the maximal utilization of recycled water, thereby reducing groundwater usage [65].

### Conclusion

Analysis of CEP data from 19 sectors and GWSP data across the 4 basins, involving 17,152 grid cells revealed a profound connection between sectoral CEP and GWSP dynamics (with a high grey relational degree greater than 0.95). Emissions from the energy industry, chemical production, and petroleum processing were strongly negatively correlated with GWSP (the maximum correlation coefficient was about −0.71). Notably, an increase of 1 Yg CO_2_/km^2^ per second in petroleum processing emissions was associated with a 4.1-mm decrease in GWSP. Special attention should be paid to the emissions from these sectors. Interestingly, agriculture and aviation showed positive correlations with GWSP in the 4 basins, and this influence lasted for up to 7 years. This suggests that certain human activities can have beneficial effects on groundwater reserves, possibly due to practices that enhance groundwater recharge or mitigate other forms of consumption. Further analysis of the spatial distribution indicated that regions with high aviation CEP tended to exhibit marginal benefits for GWSP. This suggests that regions characterized by high-quality economic development contribute to GWSP stability. Nevertheless, our results may still contain uncertainties and may remain basin dependent and data dependent. Hence, similar studies should be conducted to assess the joint impacts and importance of human activities, climate, and geology on global groundwater resources.

The impact of CEP on GWSP constitutes an important channel in the groundwater–CE–human relationship, which was previously uncertain. This study significantly advances our understanding of the relationship between human activity, quantified through CE, and the dynamics of GWS. This study contributes to an important gap in hydrological research—which has primarily focused on natural factors or several sectors—and introduces a method for quantifying the impact of human activities on GWS. This insight will enable stakeholders to identify targeted strategies for groundwater protection and management to balance human developmental needs with groundwater sustainability.

## Materials and Methods

### Study area

This study selected the YRB, PRB, GLB, and RB as the research areas, as this study aims to explore the impact of human activities on groundwater reserves. Therefore, to minimize the effects of climate change, the criterion for selecting the study areas was that the basins should have a small range of latitudinal variations (Fig. S1).

The YRB, located in the subtropical region, spans from 24°30′ to 35°45′ N latitude and from 90°33′ to 122°25′ E longitude. It features a typical monsoon climate with abundant water resources [66]. The YRB is a crucial hub for grain and energy production in China and ranks as one of the most important socioeconomic regions, holding the title of the world’s most populous river basin [67]. The PRB, located in the tropical and subtropical monsoon climate zones, is situated between 21°31′ to 26°49′ N latitude and 102°14′ to 115°53′ E longitude and enjoys abundant rainfall. With its gross domestic product (GDP) ranking second only to YRB, it is a major transportation hub and one of the most developed regions in China. The GLB, located in the northeastern United States and southern Canada, encompasses 5 major lakes: Lake Superior, Lake Michigan, Lake Huron, Lake Erie, and Lake Ontario. It is characterized by a temperate continental climate and is home to the largest surface freshwater system globally [68]. The GLB’s economy is substantial, making up about one-third of the US economy, and is particularly noted for its important manufacturing sector [69]. The RB, originating in the Alps and flowing into the North Sea, experiences considerable rainfall variations, supported by a dense river network. It is one of the most densely populated regions in Europe, highly industrialized, and hosts important industrial bases.

### CE data

Our primary source of CE data was the EDGAR v6.0 dataset [70]. We used annual CE data for the historical period from 2003 to 2018. We measured the annual average CEP as the annual average CE divided by the annual average population, which was a better measure of human activity and would help to provide a clearer explanation of the impact of CE on groundwater from a long-term perspective. To match other variables, we resampled these data to 0.5° × 0.5° grids. In addition, we removed any grids that contained missing data.

### Groundwater data

We obtained the GWS data from the GLDAS (Global Land Data Assimilation System) dataset [71] and used daily GWS for the historical period from 2003 to 2018. This was done to ensure alignment with the CE grid. Using the GWS and population data obtained, we calculated the annual GWSP for each grid. To match other variables, we resampled these data to 0.5° × 0.5° grids.

### Population data

We derived our population data from the LandScan dataset [72] and used annual population counts for the historical period from 2003 to 2018. To match other variables, we resampled these data to 0.5° × 0.5° grids.

### Regression models

We applied 2-factor fixed-effects panel regression models to estimate the impact of CEP on GWSP. This method can control for unobserved heterogeneity across regions and over time, which is critical in a context where regional development, policies, and environmental conditions can significantly influence GWSP. Using the CEP level value allowed us to interpret the regression coefficients as estimates of the change in the GWSP level value for each unit change in the explanatory variable. In its simplest form, the regression model is expressed as follows:

$${\text{GWSP}}_{g,y}=\alpha_{1}C_{g,y}+\mu_{g}+\eta_{y}+\varepsilon_{g,y}$$ (1)

where GWSP_*g*,*y*_ is the GWSP of grid cell *g* in year *y*, *C*_*g*,*y*_ is the annual average emissions in that year, and *μ_g_* and *η_y_* are regional and yearly fixed effects, respectively; *ε*_*g*,*y*_ is the region-year error. Regional fixed effects were regional dummy variables that account for unobserved, time-invariant differences between regions, such as development, policies, and cultural differences. This approach avoided the problem of omitted-variable bias that arises in interregional comparisons. Year fixed effects acted as global dummy variables for each year, accounting for contemporaneous shocks to both CEP and GWSP data in the basin, such as urbanization or climate change. Finally, for our main modeling specification, we added a series of CEP analogies:

$$\begin{array}{c} {\text{GWSP}}_{g,y}=\alpha_{1}C_{1,g,y}+\alpha_{2}C_{2,g,y}+,\ldots ,\\ \;+\alpha_{n}C_{n,g,y}+\mu_{g}+\eta_{y}+\varepsilon_{g,y} \end{array}$$ (2)

and we added the population to our model as a comparison (details in Tables S2 to S5):

$$\begin{array}{c} {\text{GWSP}}_{g,y}=\alpha_{1}C_{1,g,y}+\alpha_{2}C_{2,g,y}+,\ldots ,\\ \;+\alpha_{n}C_{n,g,y}+\alpha_{n+1}P_{g,y}+\mu_{g}+\eta_{y}+\varepsilon_{g,y} \end{array}$$ (3)

where *C*_*n*, *g*,*y*_ describes the CEP of each sector of grid cell *g* in year *y* and *P*_*g*,*y*_ is the annual population in that year. To explore the persistence effect of CEP on GWSP, we also considered the impact of the lag of CEP on GWSP in further model variations:

$$\begin{array}{c} {\text{GWSP}}_{g,y}=\alpha_{1}C_{1,g,y-1}+\alpha_{2}C_{2,g,y-1}+,\ldots ,\\ \;+\alpha_{n}C_{n,g,y-1}+\mu_{g}+\eta_{y}+\varepsilon_{g,y} \end{array}$$ (4)

$$\begin{array}{c} {\text{GWSP}}_{g,y}=\alpha_{1}C_{1,g,y-2}+\alpha_{2}C_{2,g,y-2}+,\ldots ,\\ \;+\alpha_{n}C_{n,g,y-2}+\mu_{g}+\eta_{y}+\varepsilon_{g,y} \end{array}$$ (5)

$$\begin{array}{c} {\text{GWSP}}_{g,y}=\alpha_{1}C_{1,g,y-7}+\alpha_{2}C_{2,g,y-7}+,\ldots ,\\ \;+\alpha_{n}C_{n,g,y-7}+\mu_{g}+\eta_{y}+\varepsilon_{g,y} \end{array}$$ (6)

where *C*_*n*,*g*,*y*−*b*_ represents the CEP’s lagged *b* years relative to GWSP. To investigate the impact of interannual and interdecadal changes in CEP on GWSP, we measured the interannual variability as the difference between the annual average CEP and the previous year’s annual average CEP to observe the variability of the impact of CE on groundwater from a short-term variability perspective, and we created a new model variation using advanced data allocation:

$$\begin{array}{c} \Delta {\text{GWSP}}_{g,y}=\alpha_{1}\Delta C_{1,g,y}+\alpha_{2}\Delta C_{2,g,y}+,\ldots ,\\ \;+\alpha_{n}\Delta C_{n,g,y}+\mu_{g}+\eta_{y}+\varepsilon_{g,y} \end{array}$$ (7)

where Δ*C*_*n*,*g*,*y*_ and Δ*GWSP*_*g*,*y*_ describe the growth of CEP and GWSP in the sample area relative to the previous year.

### Marginal effects

The marginal effect of CEP was the change in GWSP estimated to result from an increase of 1 Yg CO_2_/km^2^ per second in CEP. This was calculated as the first derivative of the change or growth in GWSP with respect to the growth in CEP, given by the equation describing the specifications of the regression model. For example, for the specification shown in Eq. 1, the marginal effect would simply be the constant *α*_1_. Therefore, the impact of an increase in one unit in CEP depends on *α*_1_.

### Evaluation criterion

The above predicted values were compared with the corresponding observations, and the differences were assessed using the adjusted *R*^2^ and AIC. The computational formulae for the adjusted *R*^2^ and AIC are:

$$\text{Adjusted}\;R^{2}=1-\frac{\left(1-R^{2}\right)\left(N-1\right)}{N-n-1}$$ (8)

where *R*^2^ is the coefficient of determination, *N* is the sample size of GWSP prediction data, and *n* is the number of CE sectors included in the panel model.

$$R^{2}=1-\frac{\Sigma_{g=1}^{s}\Sigma_{y=1}^{m}{\left({\hat{\mathrm{GWSP}}}_{g,y}-\mathrm{GWS}P_{g,y}\right)}^{2}}{\Sigma_{g=1}^{s}\Sigma_{y=1}^{m}\left({\bar{\text{GWSP}}}_{g,y}-\mathrm{GWS}P_{g,y}\right)}$$ (9)

where ${\hat{\mathrm{GWSP}}}_{g,y}$ represents the predicted GWSP for grid cell *g* in year *y*, while ${\bar{\text{GWSP}}}_{g,y}$ denotes the average GWSP. The *m* represents the number of years *y*, and *N* = *S* * *m*, where *S* signifies the number of grid cells.

$$\text{AIC}=2k-2\text{ln}\left(L\right)$$ (10)

where *k* denotes the number of parameters in the panel model and *L* represents the maximum likelihood of the panel model.

### Grey relational analysis

The grey relational analysis method was used to assess the connection and importance ranking of the relationship between various sectors of CEP and GWSP [73]. First, CEP and GWSP data were standardized to eliminate scale differences between the different variables. Here, we performed a normalization process, and the formulae were as follows:

$${\text{CEP}}_{i}\left(k\right)=\frac{{\text{CEP}}_{i}^{\prime }\left(k\right)}{\frac{1}{s}\sum_{k=1}^{s}{\text{CEP}}_{i}^{\prime }\left(k\right)}$$ (11)

$$\text{GWSP}\left(k\right)=\frac{{\text{GWSP}}^{\prime }(k)}{\frac{1}{s}\sum_{k=1}^{s}{\text{GWSP}}^{\prime }(k)}$$ (12)

where ${\text{CEP}}_{i}^{\prime }\left(k\right)$ represents the CEP of the *k* sample for the *i* emissions sector, where *i* = 1, 2, ..., *n* and *k* = 1, 2, ..., *s*. The $\frac{1}{s}\sum_{k=1}^{s}{\text{CEP}}_{i}^{\prime }\left(k\right)$ denotes the mean value of CEP′, and CEP*_i_*(*k*) represents the value of the CEP after normalization processing.

Next, we calculated the grey relational coefficient, which was used to measure the connection between different sectors and GWSP. The calculation formula was as follows:

$$\begin{array}{c} \zeta_{i}\left(k\right)=\\ \frac{\underset{i}{\text{min}}\underset{k}{\text{min}}\left|\text{GWSP}\left(k\right)-{\text{CEP}}_{i}\left(k\right)\right|+\rho \cdot \underset{i}{\text{max}}\underset{k}{\text{max}}\left|\text{GWSP}\left(k\right)-{\text{CEP}}_{i}\left(k\right)\right|}{\left|\text{GWSP}\left(k\right)-{\text{CEP}}_{i}\left(k\right)\right|+\rho \cdot \underset{i}{\text{max}}\underset{k}{\text{max}}\left|\text{GWSP}\left(k\right)-{\text{CEP}}_{i}\left(k\right)\right|} \end{array}$$ (13)

where *ζ_i_*(*k*) represents the grey relational coefficient, GWSP(*k*) represents the standardized value of GWSP for sample *k*, and |GWSP(*k*) − CEP(*k*)| represents the absolute difference between the CEP sequence of various sectors and the corresponding sample in the GWSP sequence. The *ρ* is the resolution coefficient, typically taking values within the range of 0 to 1 and is commonly set to *ρ* = 0.5 [74–77].

Then, we calculated the grey relational degree, which represents the degree of connection between CEP and GWSP. The formula was as follows:

$$c_{0i}=\frac{1}{s}\Sigma_{k=1}^{s}\zeta_{i}\left(k\right)$$ (14)

where *c*_0*i*_ is the overall grey relational degree, grey relational degrees typically ranged from 0 to 1, with larger degrees indicating stronger connections, and if they were the same, the grey relational degree equaled 1.

Finally, the sectors were ranked on the basis of the magnitude of their grey relational degrees to identify sectors with higher connections. A higher grey relational degree indicates a stronger connection between CEP and GWSP in these sectors.

### Statistical analysis

A normality test was performed on the data for each basin using the obtained emissions and groundwater datasets. On the basis of the results of the normal distribution test, we analyzed the Spearman rank correlation between CEP and GWSP using R v. 4.1.2. The formula was as follows:

$$r=1-\frac{6\sum d_{k}^{2}}{s\left(s^{2}-1\right)}$$ (15)

where *r* is the Spearman rank correlation coefficient, *r* typically ranged from −1 to 1, with larger |*r*| indicating stronger correlation, *d_k_* is the difference between the GWSP and CEP rankings of the corresponding grid, and *s* is the number of data pairs.

## Data Availability

The CE data are sourced from the European Commission, Joint Research Centre (https://edgar.jrc.ec.europa.eu/dataset_ghg60). The GWS data are obtained from NASA’s GLDAS-2 (https://disc.gsfc.nasa.gov/datasets/GLDAS_CLSM025_DA1_D_2.2/summary). The population data are derived from the Oak Ridge National Laboratory LandScan Program (https://landscan.ornl.gov/). The code developed in the current study is available from the corresponding author on reasonable request.
