# Supplementary material for: How Human Activities Affect Groundwater Storage
Source: Research (Wash D C). 2024 May 29;7:0369. doi: 10.34133/research.0369 (PMC11134413; doi:10.34133/research.0369)
Supplement: Supplementary 1 — Figs. S1 to S4 Tables S1 to S5 [file research.0369.f1.zip › Supplementary_Materials.docx]

**Supplementary Materials for:**

**How Human Activities Impact Groundwater Storage?**

Ying Zhao^1,2†^, Meiling Zhang^1,2†^, Zhuqing Liu^1,2†^, Jiabin Ma^1,2^, Fan Yang^1,2^*, Huaming Guo^3^, Qiang Fu^1,2^*

**Affiliations**

^1.^ School of Water Conservancy & Civil Engineering, Northeast Agricultural University, Harbin 150030, China.

^2.^ International Cooperation Joint Laboratory of Health in Cold Region Black Soil Habitat of the Ministry of Education, Harbin 150030, China.

^3.^ Ministry of Education Key Laboratory of Groundwater Circulation and Environmental Evolution & School of Water Resources and Environment, China University of Geosciences (Beijing), Beijing 100083, China.

* Address correspondence to: [yangfan_neau@163.com](mailto:yangfan_neau@163.com) and [fuqiang@neau.edu.cn](mailto:fuqiang@neau.edu.cn)

† These authors contributed equally to this work.

**Content**

**Table S1.** Abbreviations table

**Table S2.** Regression results from different panel-regression-model specifications for CEP on GWSP of the YRB

**Table S3.** Regression results from different panel-regression-model specifications for CEP on GWSP of the PRB

**Table S4.** Regression results from different panel-regression-model specifications for CEP on GWSP of the GLB

**Table S5.** Regression results from different panel-regression-model specifications for CEP on GWSP of the RB

**Fig. S1.** Map of the study areas: the YRB, PRB, GLB, and RB.

**Fig. S2.** Heatmap of correlation analysis for various sectors CE and GWS in four basins.

**Fig. S3a.** Spatial distribution of total CEP and GWSP in the YRB from 2003 to 2018.

**Fig. S3b.** Spatial distribution of total CEP and GWSP in the PRB from 2003 to 2018.

**Fig. S3c.** Spatial distribution of total CEP and GWSP in the GLB from 2003 to 2018.

**Fig. S3d.** Spatial distribution of total CEP and GWSP in the RB from 2003 to 2018.

**Fig. S4a.** Distribution of the marginal effects of CEP on GWSP in YRB.

**Fig. S4b.** Distribution of the marginal effects of CEP on GWSP in PRB.

**Fig. S4c.** Distribution of the marginal effects of CEP on GWSP in GLB.

**Fig. S4d.** Distribution of the marginal effects of CEP on GWSP in RB.

**Table S1.** Abbreviations table

| Index | Abbreviations | Full name |
| --- | --- | --- |
| Main abbreviations | CE | Carbon emissions |
|  | CEP | Carbon emissions per capita |
|  | GHG | Greenhouse gas |
|  | GWS | Groundwater storage |
|  | GWSP | Groundwater storage per capita |
| Basin | YRB | Yangtze River Basin |
|  | PRB | Pearl River Basin |
|  | GLB | Great Lakes Basin |
|  | RB | Rhine Basin |
| Emission sectors | AGS | Agricultural soils per capita |
|  | CHE | Chemical processes per capita |
|  | ENE | Energy industry per capita |
|  | IND | Combustion for manufacturing per capita |
|  | IRO | Iron and steel production per capita |
|  | NEU | Non-energy use of fuels per capita (Petroleum processing) |
|  | NFE | Production of non-ferrous metals per capita |
|  | NMM | Production of non-metallic minerals per capita |
|  | PRO | Fuel production/transmission per capita |
|  | PRU_SOL | Solvent and products use per capita (Industrial manufacture per capita) |
|  | RCO | Residential per capita |
|  | REF_TRF | Oil refineries and Transformation industry per capita |
|  | SWD_INC | Solid waste disposal per capita |
|  | TNR_A_CDS | Aviation climbing & descent per capita |
|  | TNR_A_CRS | Aviation cruise per capita |
|  | TNR_A_LTO | Aviation landing & takeoff per capita |
|  | TNR_Others | Railways, pipelines, off-road transport per capita |
|  | TNR_Ship | Shipping per capita |
|  | TRO_noRES | Road transport per capita |
|  | AFOLU | Agriculture, Forestry, and Other Land Use per capita |
|  | IPPU | Industrial Processes and Product Use per capita |
|  | TC | Total carbon emissions per capita |

**
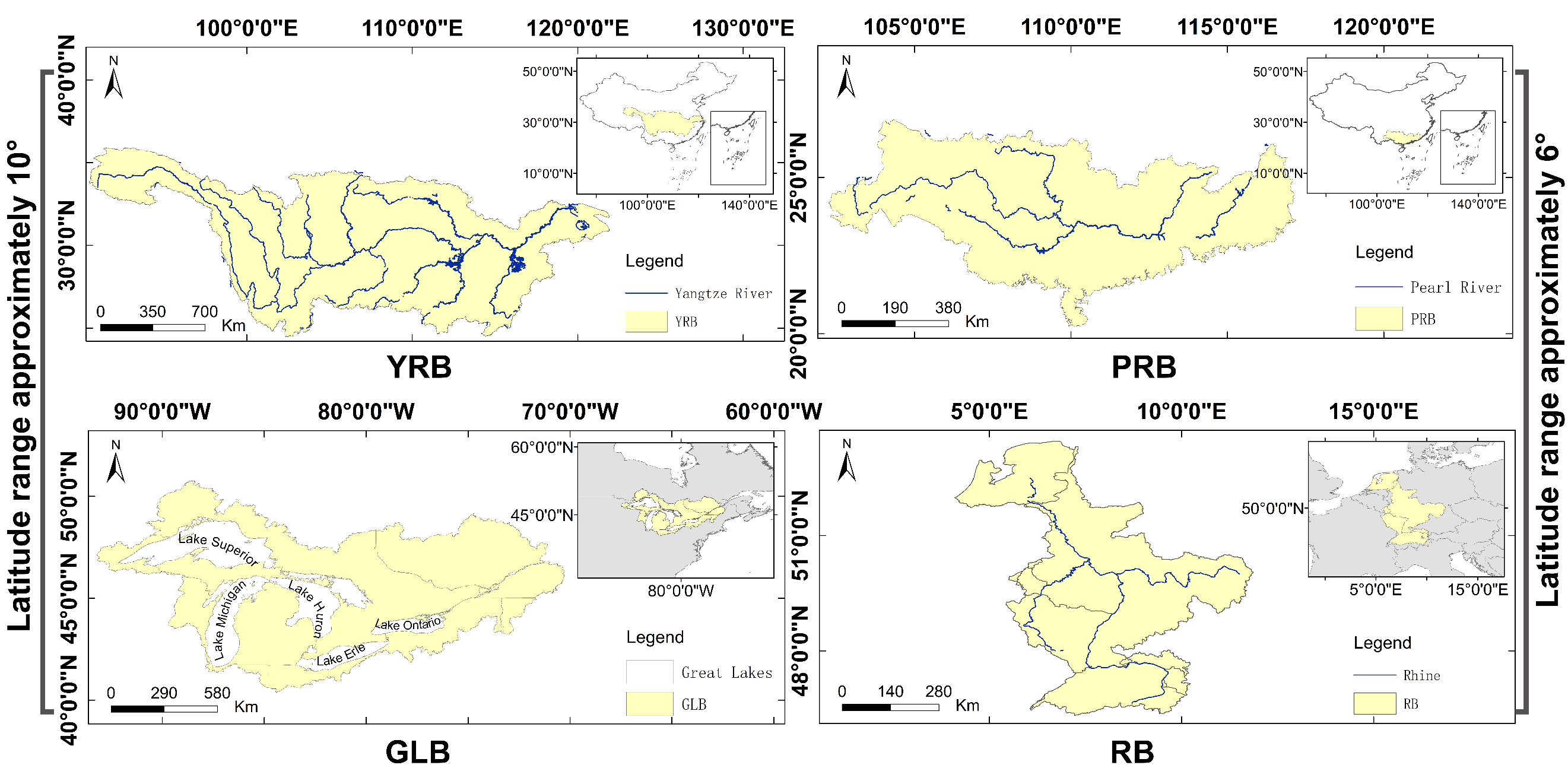
**

**Fig. S1.** Map of the study areas: the YRB, PRB, GLB, and RB.

**
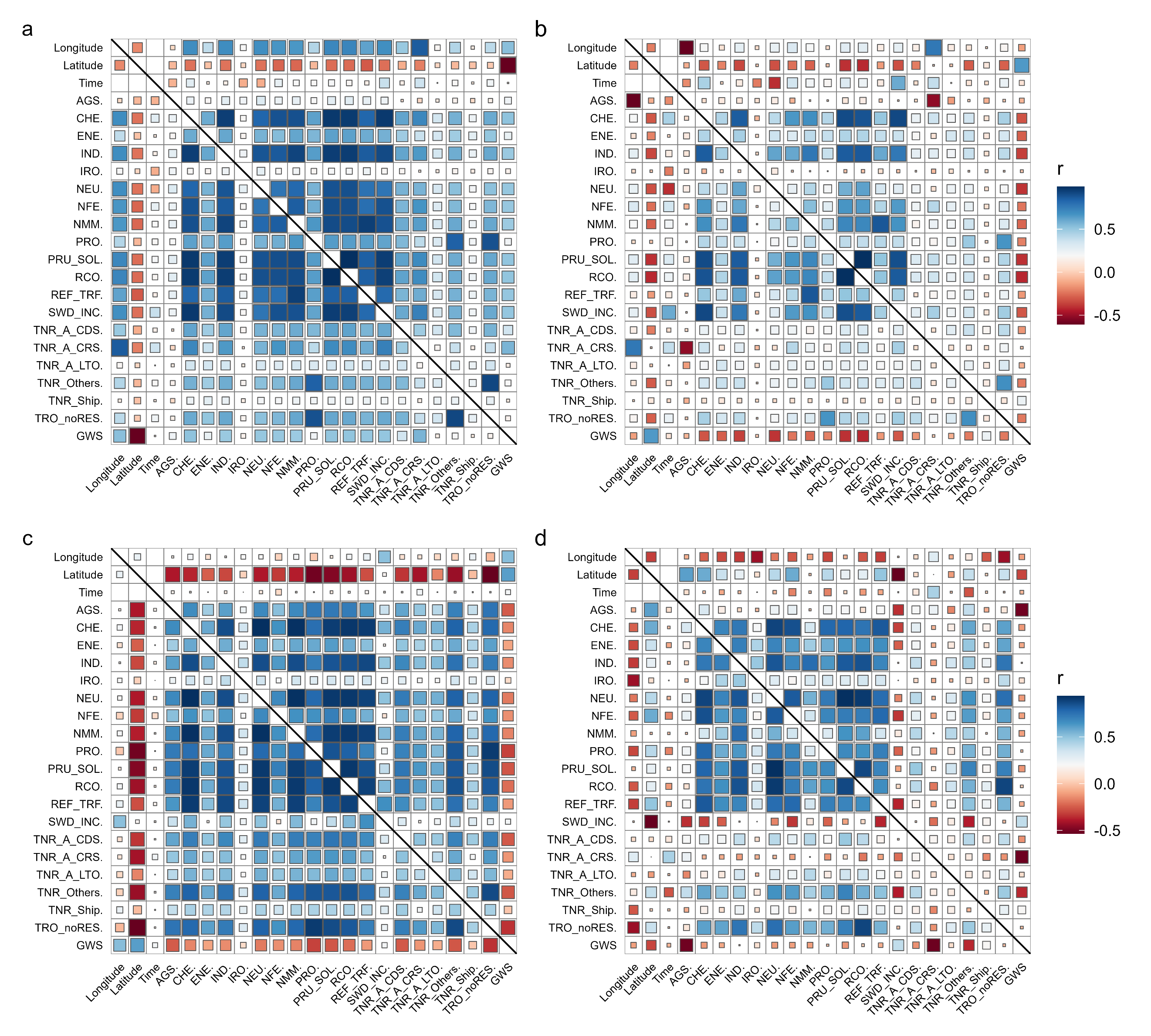
**

**Fig. S2.** Heatmap of correlation analysis for various sectors CE and GWS in four basins. a. YRB; b. PRB; c. GLB; d. RB.

**Table S2.** Regression results from different panel-regression-model specifications for CEP on GWSP of the YRB

Coefficients from four regression models are shown. Coefficients estimated effect of a one-unit change in the CEP on the GWSP change. On the basis of the adjusted R^2^ and AIC, we select ***model (4)*** as our preferred specification. **P*<0.05, ***P*<0.01, ****P*<0.001.

**Table S3.** Regression results from different panel-regression-model specifications for CEP on GWSP of the PRB

**Table S4.** Regression results from different panel-regression-model specifications for CEP on GWSP of the GLB

**Table S5.** Regression results from different panel-regression-model specifications for CEP on GWSP of the RB

**
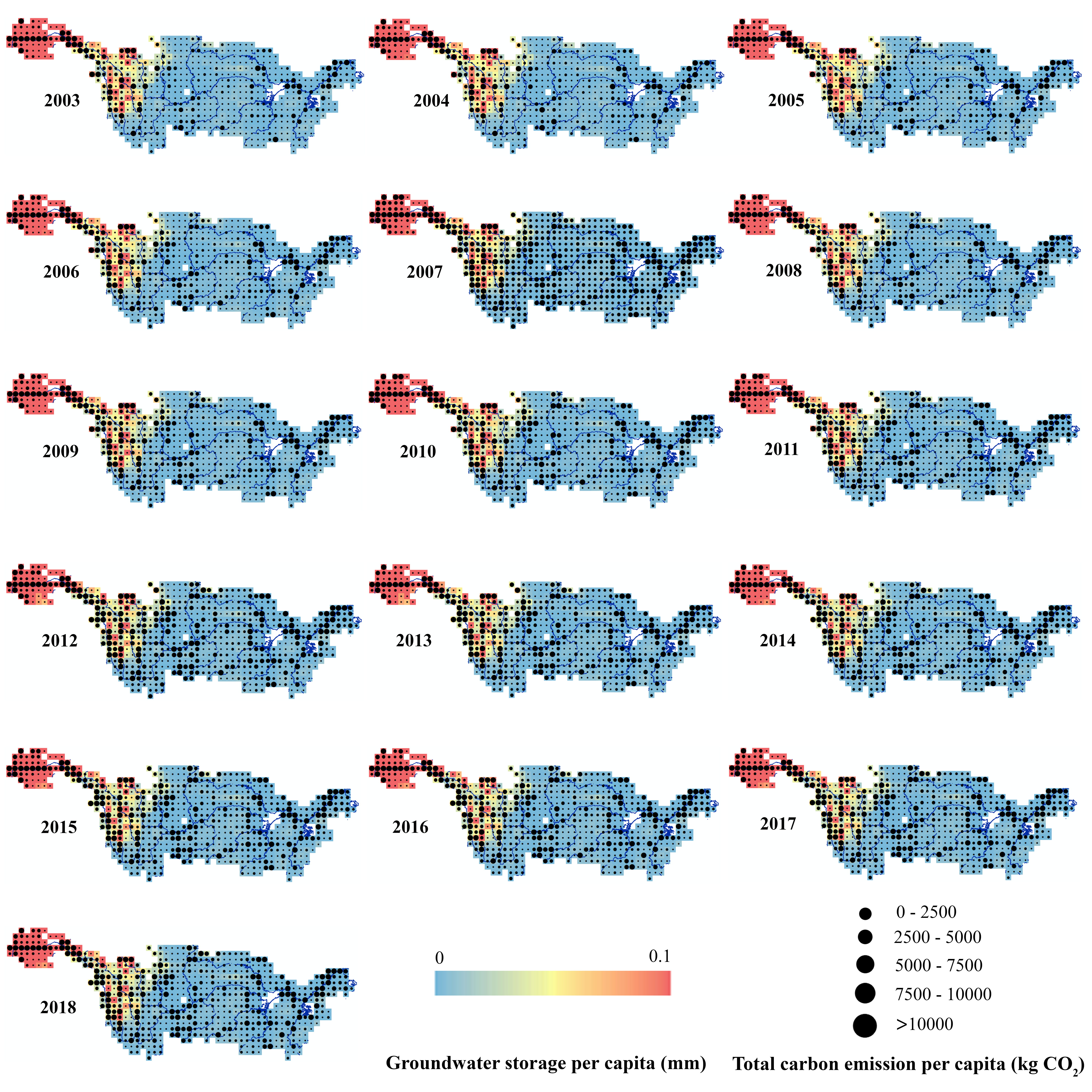
**

**Fig. S3a.** Spatial distribution of total CEP and GWSP in the YRB from 2003 to 2018.


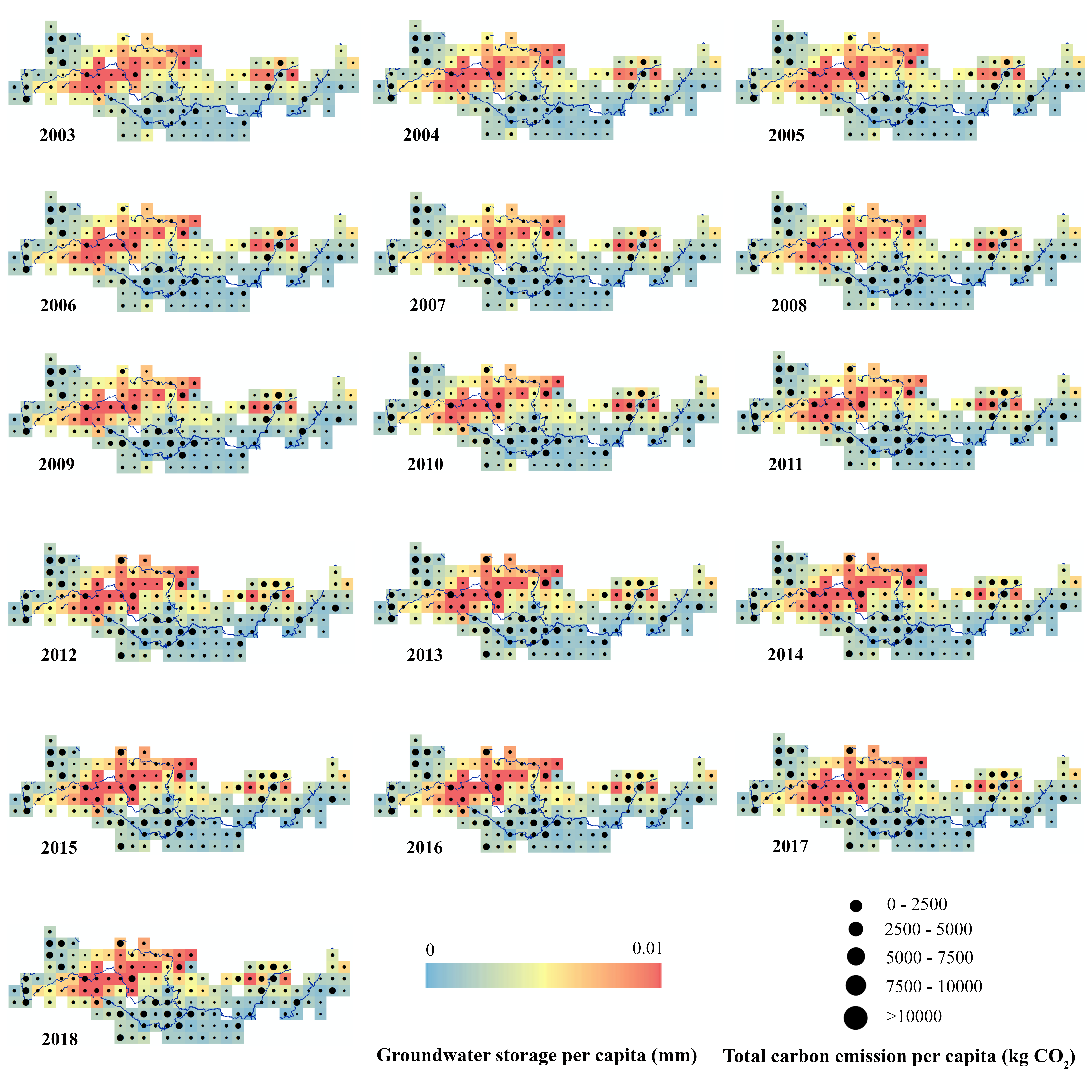


**Fig. S3b.** Spatial distribution of total CEP and GWSP in the PRB from 2003 to 2018.


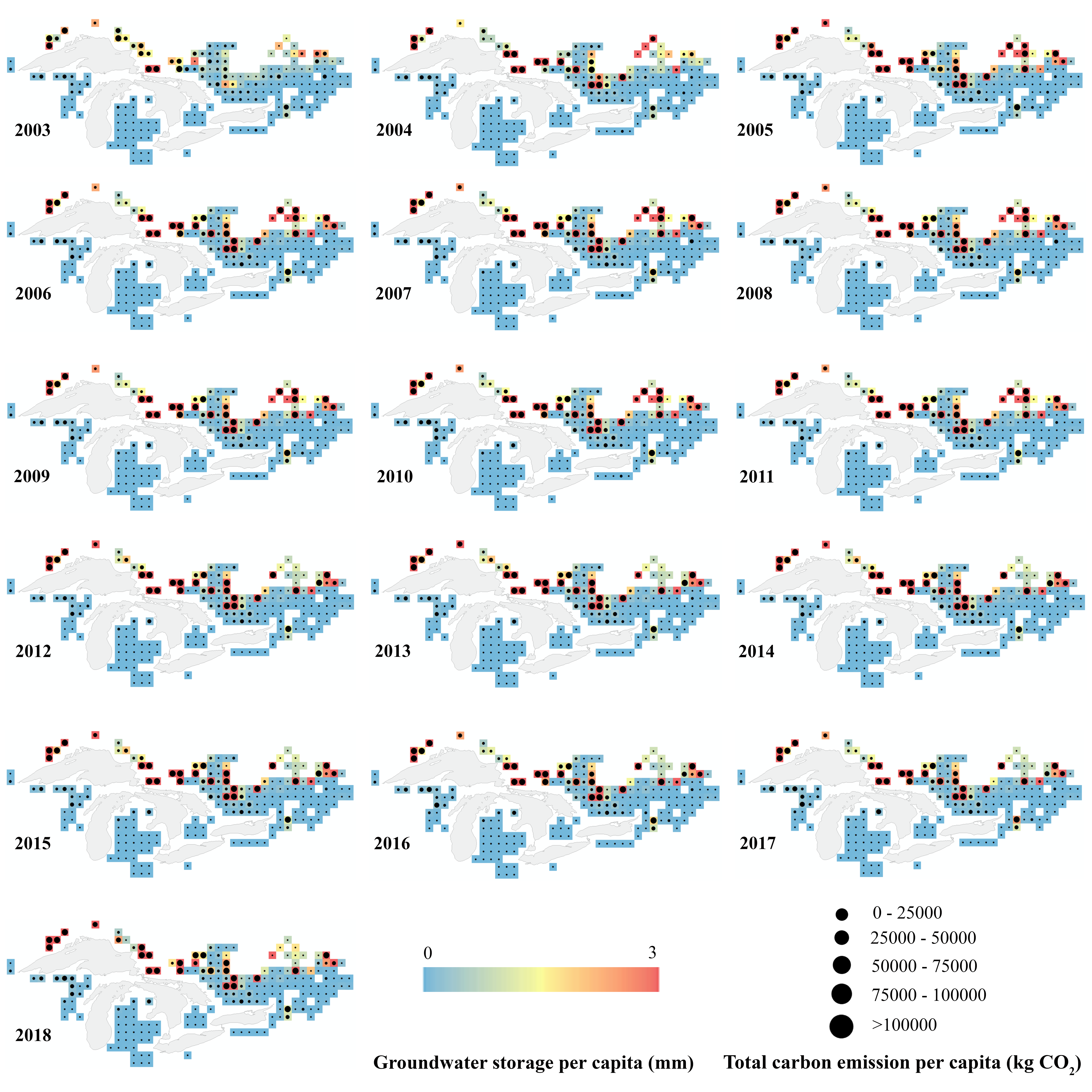


**Fig. S3c.** Spatial distribution of total CEP and GWSP in the GLB from 2003 to 2018.

**
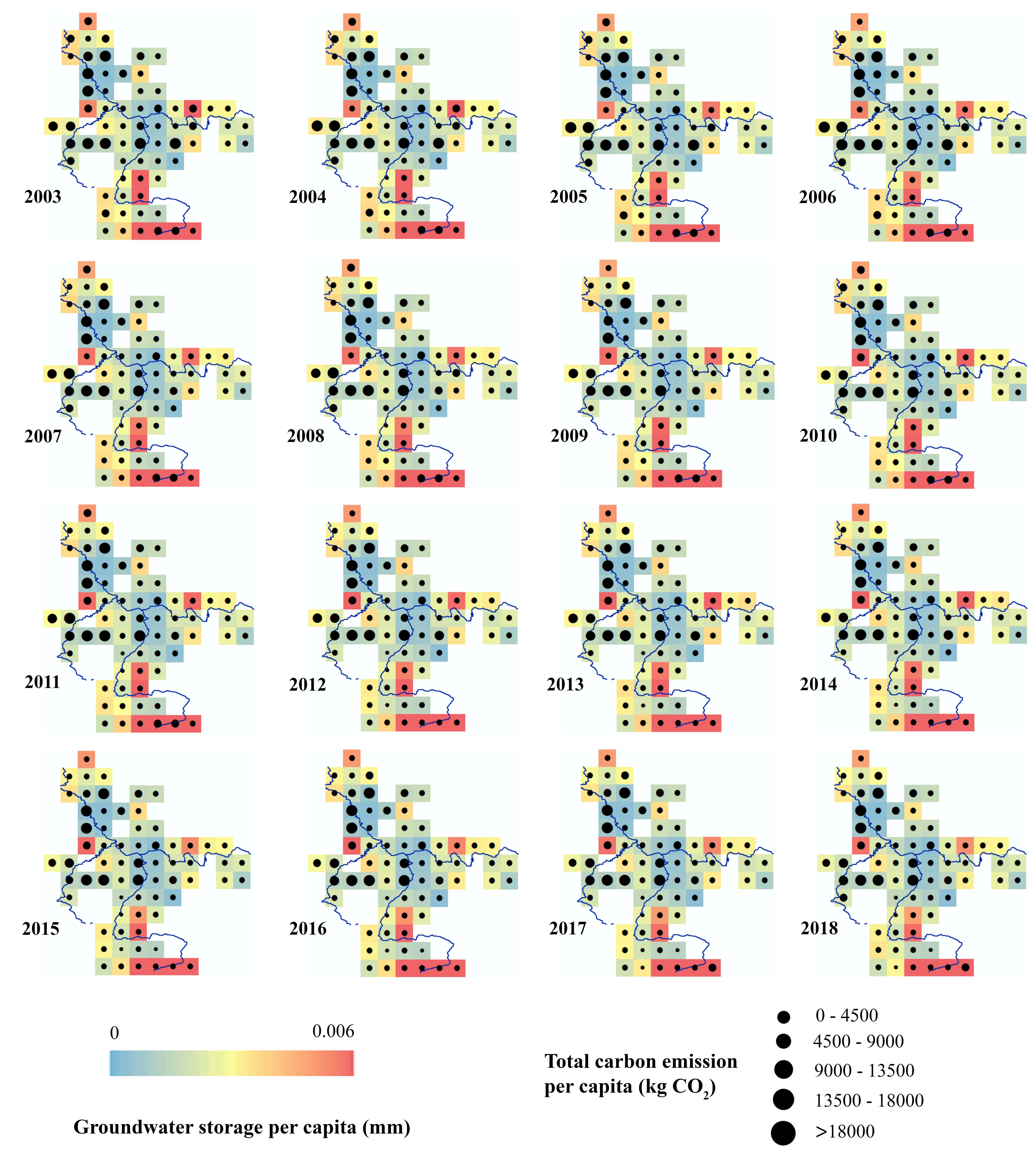
**

**Fig. S3d.** Spatial distribution of total CEP and GWSP in the RB from 2003 to 2018.

**
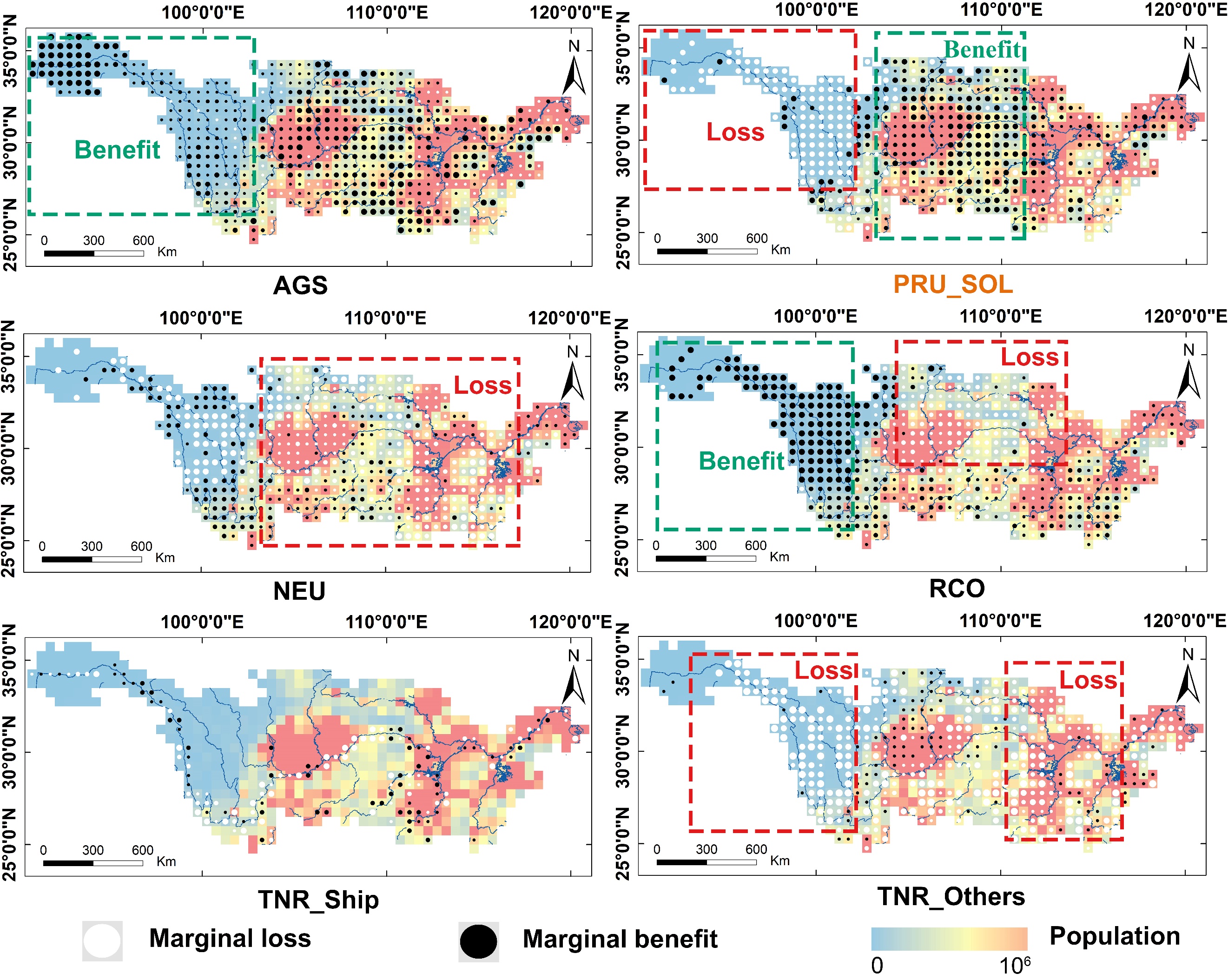
**

**Fig. S4a**. Distribution of the marginal effects of CEP on GWSP in YRB. The size of the dots indicating the strength of the marginal effects.


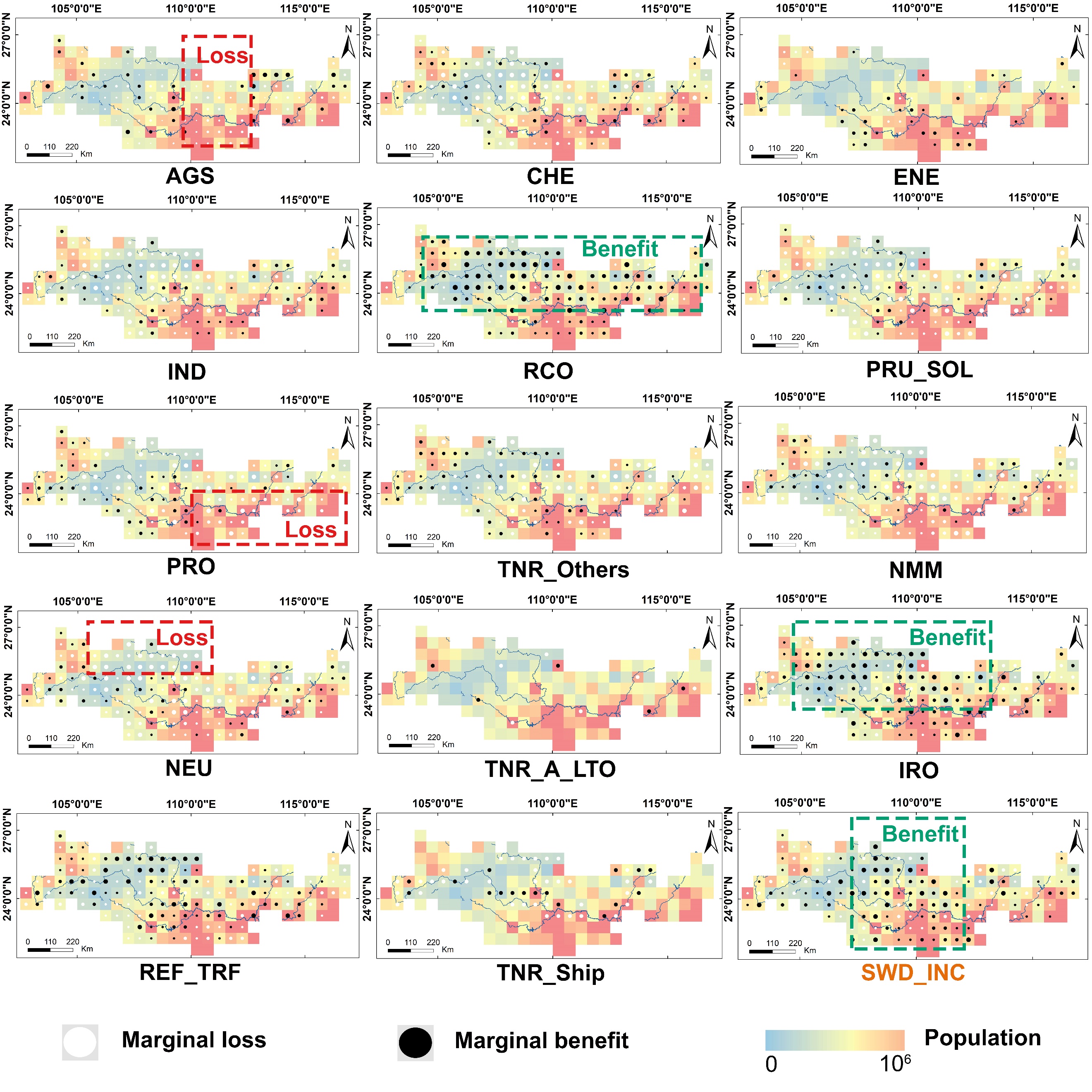


**Fig. S4b**. Distribution of the marginal effects of CEP on GWSP in PRB.


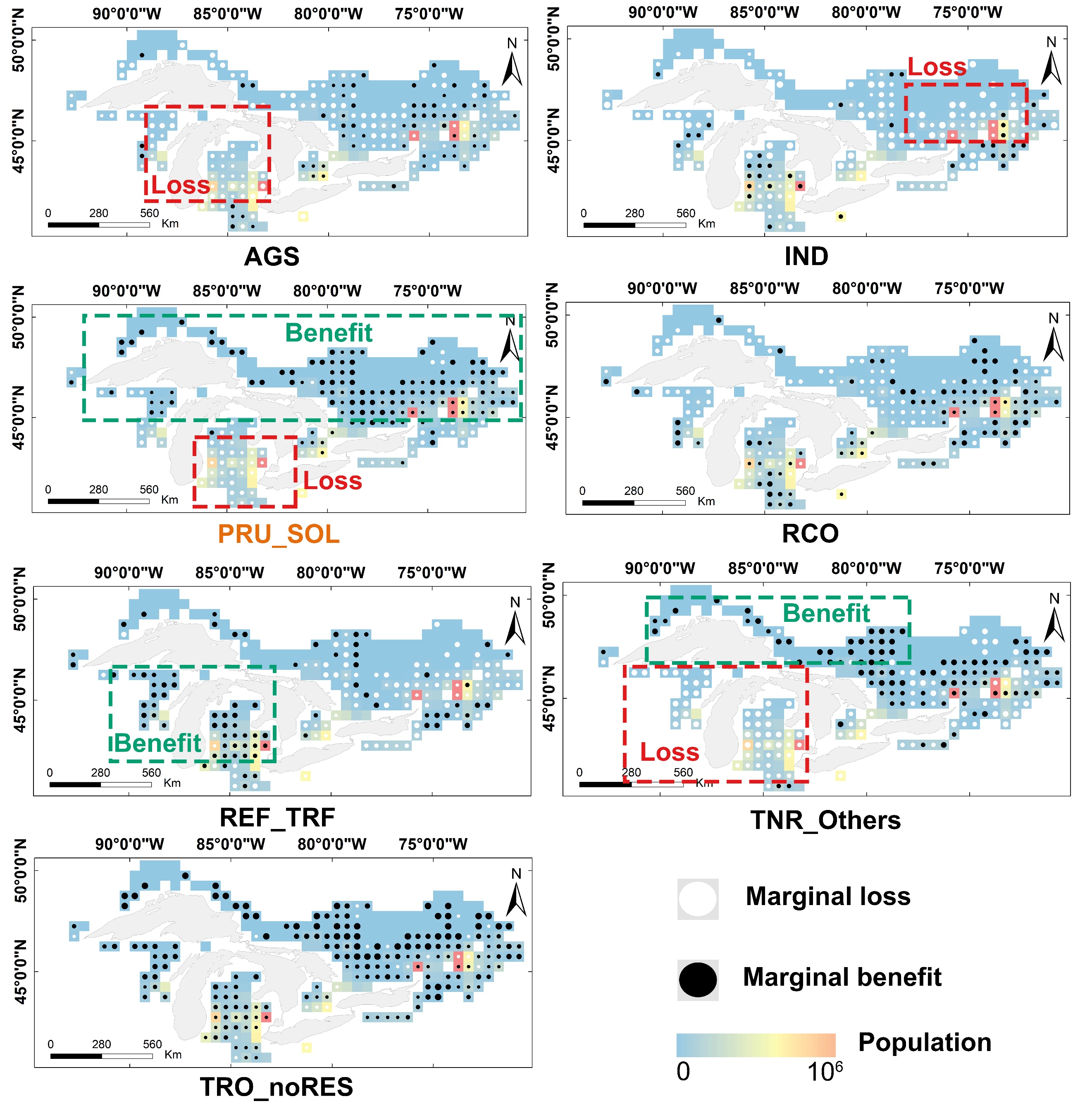


**Fig. S4c**. Distribution of the marginal effects of CEP on GWSP in GLB.


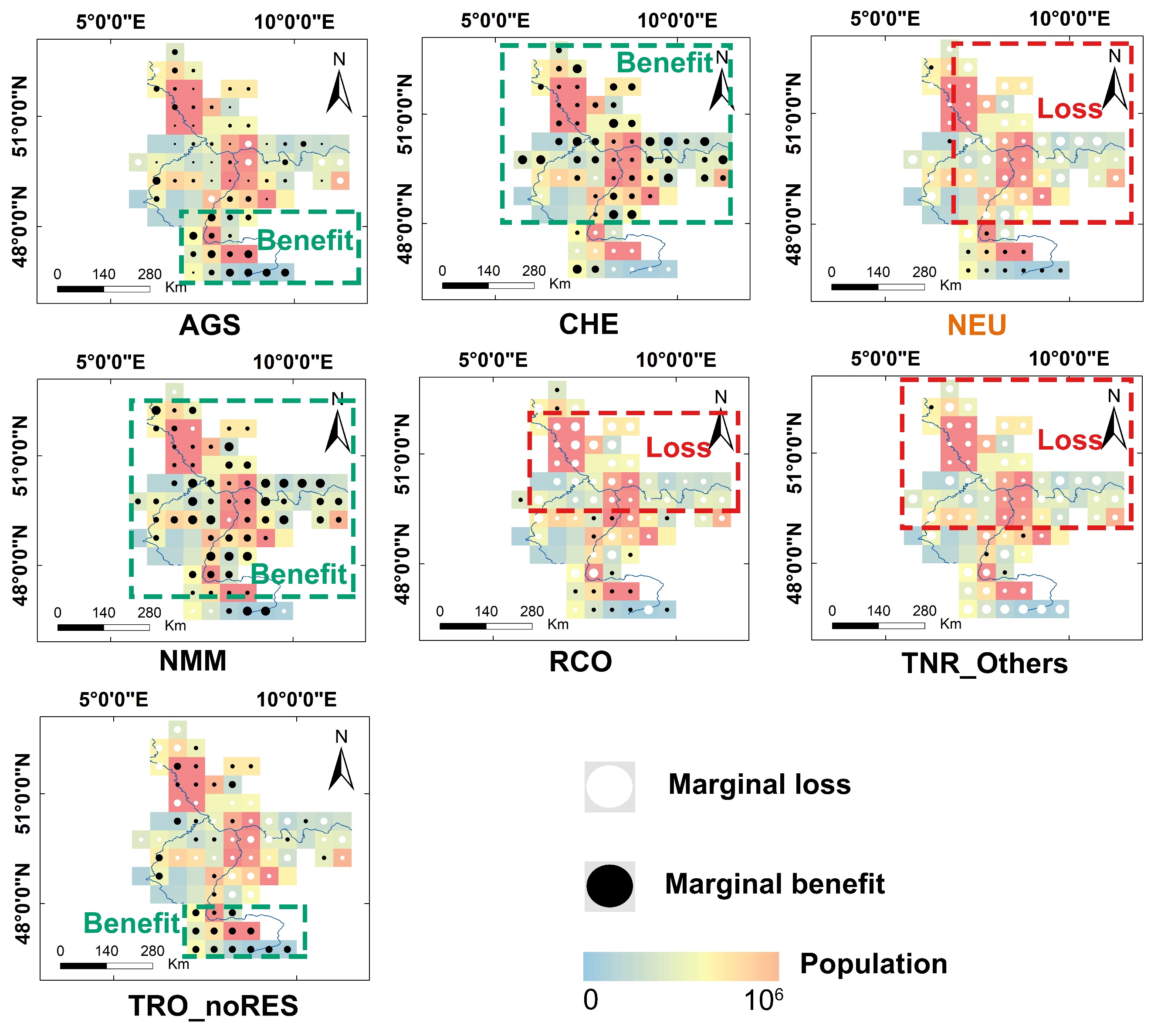


**Fig. S4d**. Distribution of the marginal effects of CEP on GWSP in RB.
