# Supplementary figures and images for: How Human Activities Affect Groundwater Storage
Source: Research (Wash D C). 2024 May 29;7:0369. doi: 10.34133/research.0369 (PMC11134413; doi:10.34133/research.0369)

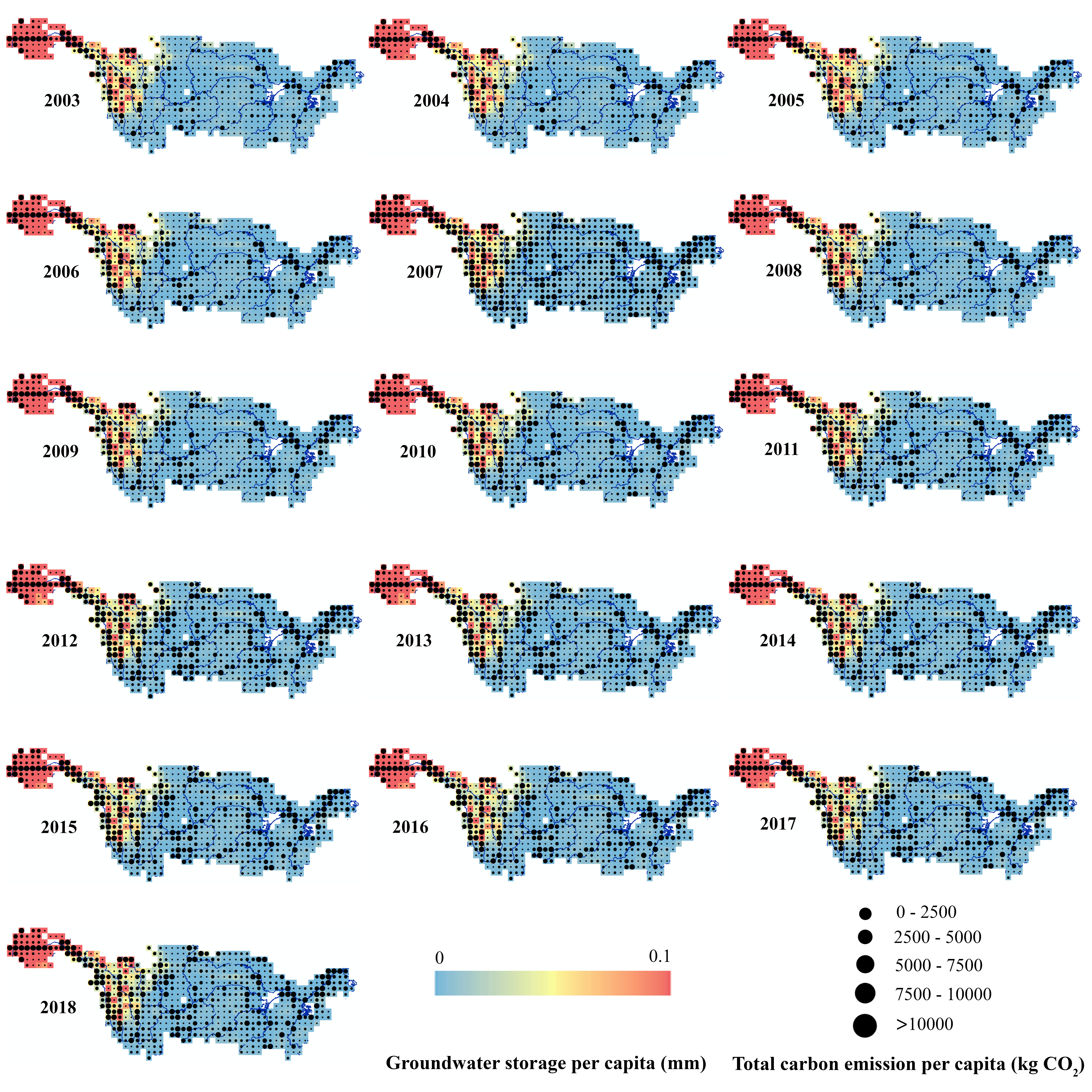

Supplement: Supplementary 1 — Figs. S1 to S4 Tables S1 to S5 [file research.0369.f1.zip › Fig. S3a.png]

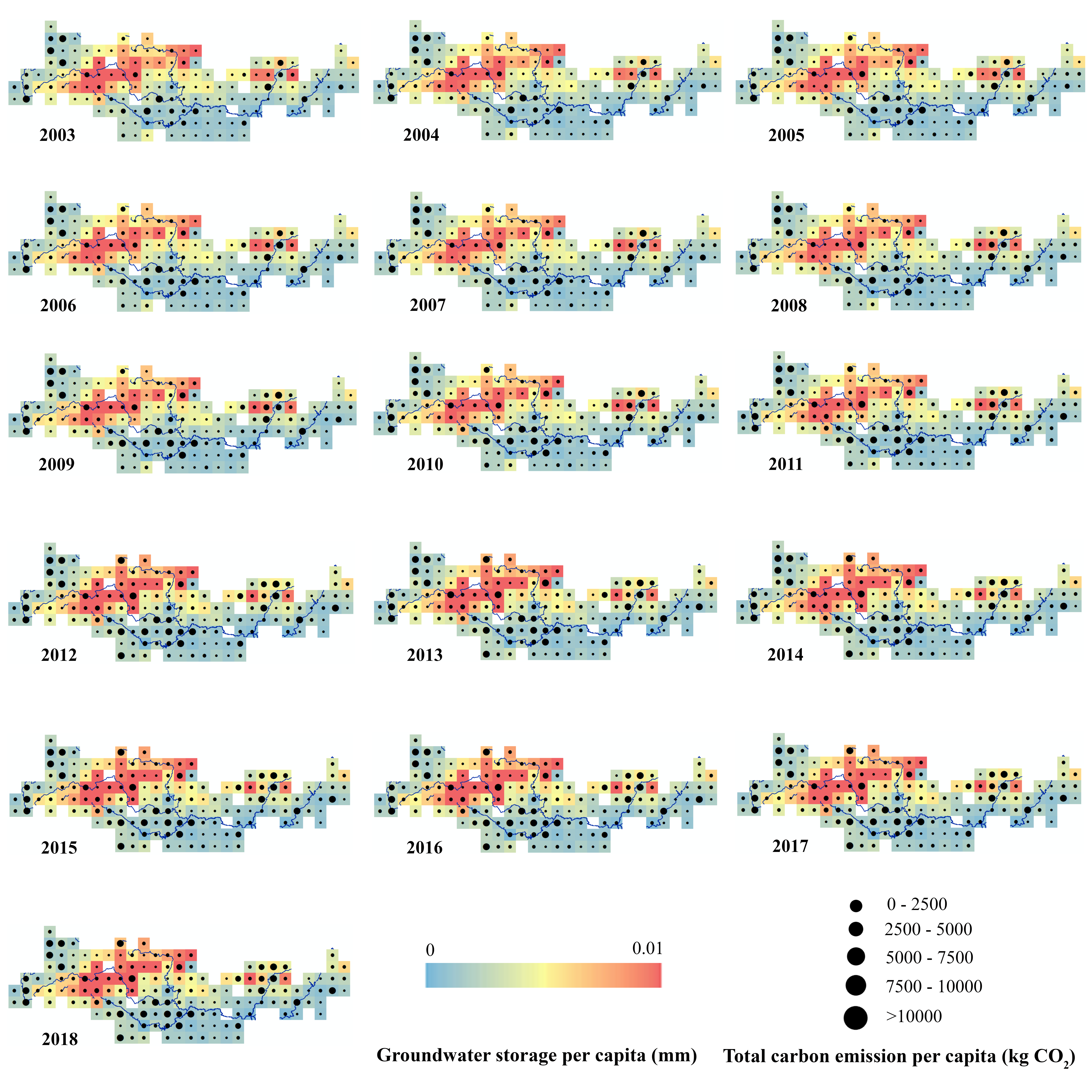

Supplement: Supplementary 1 — Figs. S1 to S4 Tables S1 to S5 [file research.0369.f1.zip › Fig. S3b.png]

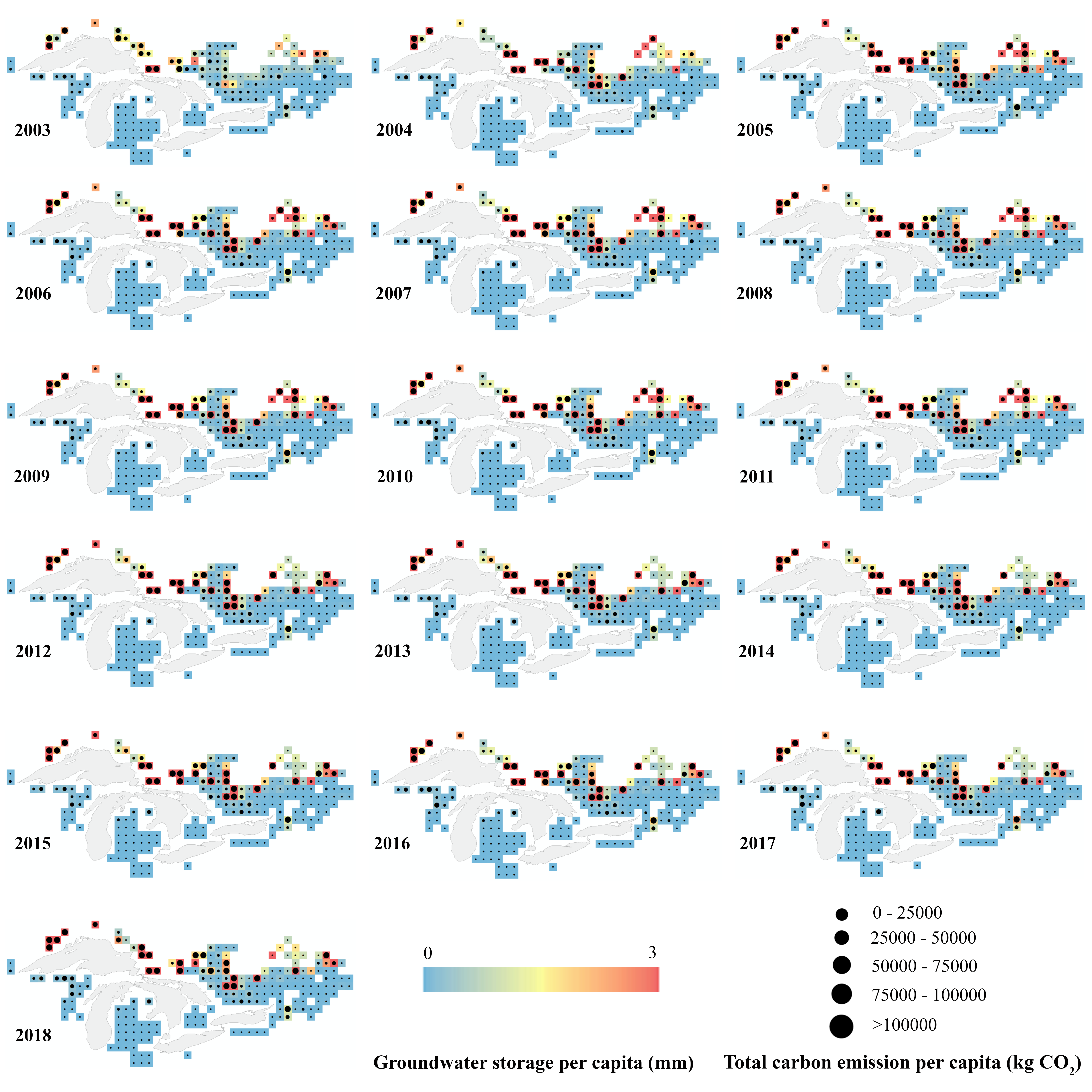

Supplement: Supplementary 1 — Figs. S1 to S4 Tables S1 to S5 [file research.0369.f1.zip › Fig. S3c.png]

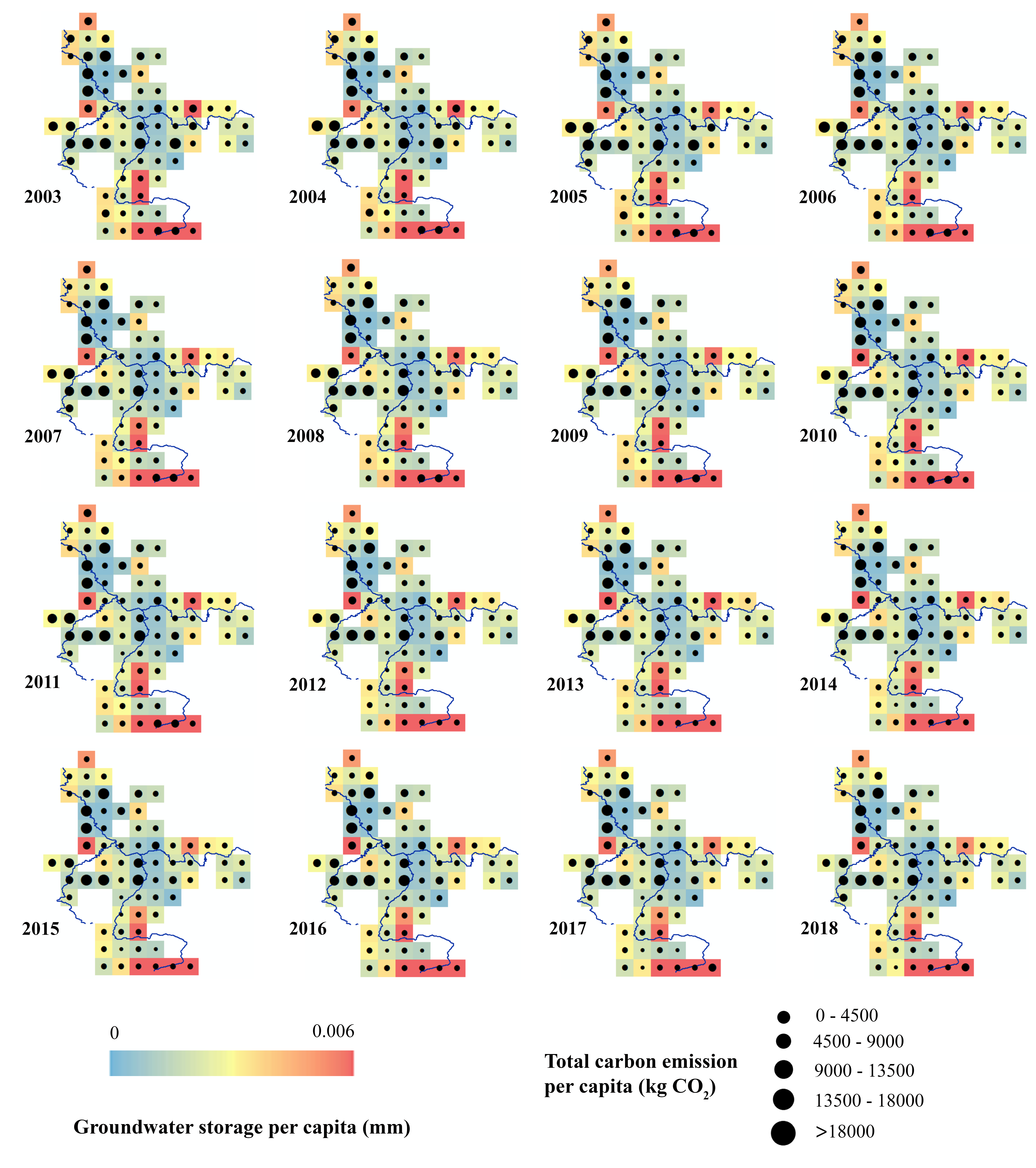

Supplement: Supplementary 1 — Figs. S1 to S4 Tables S1 to S5 [file research.0369.f1.zip › Fig. S3d.png]

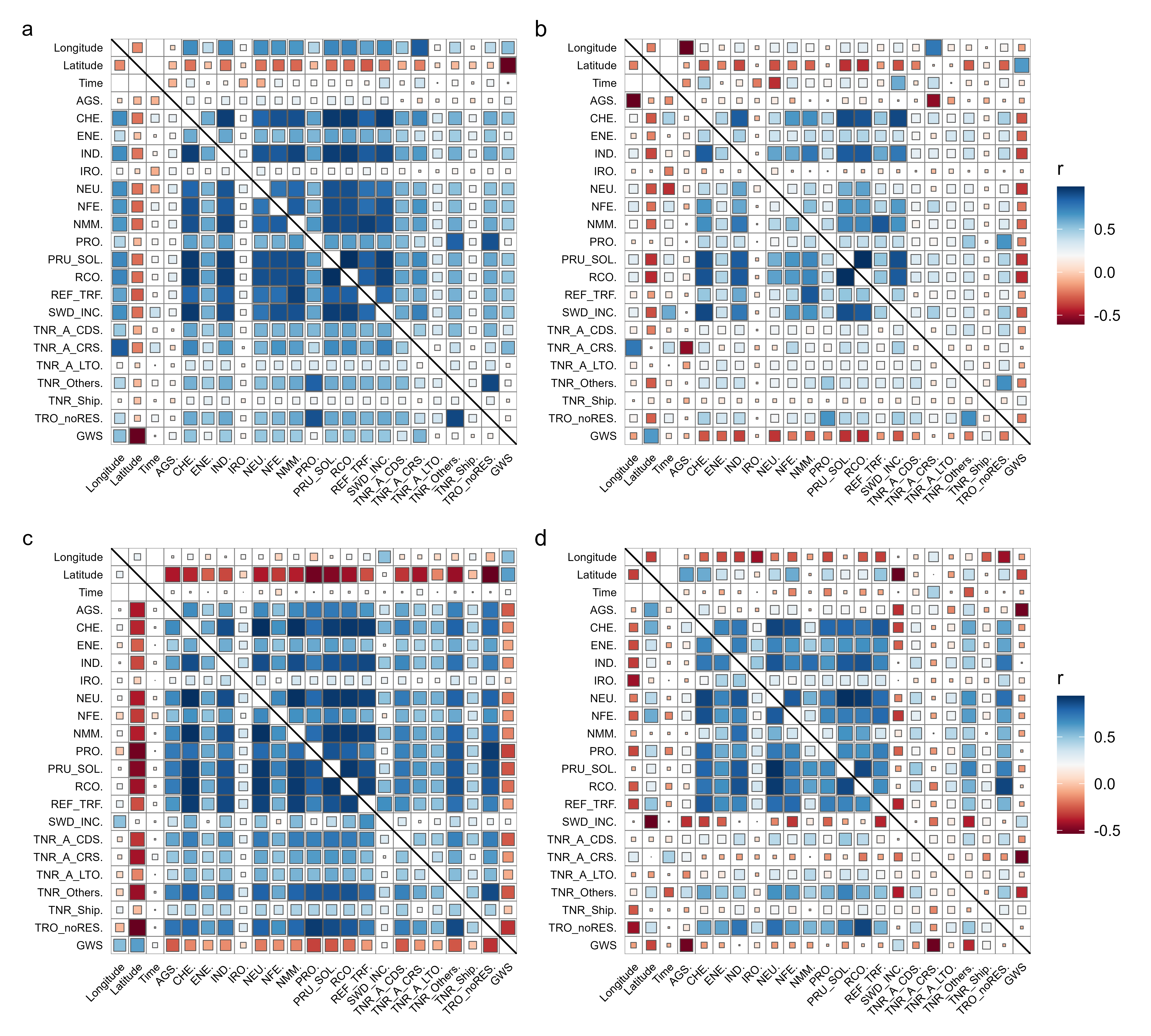

Supplement: Supplementary 1 — Figs. S1 to S4 Tables S1 to S5 [file research.0369.f1.zip › Fig S2.png]
